# The Responsibility of Selecting the Ideal Simillimum

**Published:** 1894-02

**Authors:** 


### HOMŒOPATHIC PHYSICIAN,

A MONTHLY JOURNAL OF
HOMŒOPATHIC MATERIA MEDICA AND CLINICAL MEDICINE.

"If our school ever gives up the strict inductive method of Hahnemann, we are lost, and deserve only to be mentioned as a caricature in the history of medicine."—CONSTANTINE HERING.

Vol. XIV. FEBRUARY, 1894. No. 2.

### EDITORIAL.

THE RESPONSIBILITY OF SELECTING THE IDEAL SIMILLIMUM.—An old lady, suffering from an attack of grippe, having been relieved of the most urgent symptoms, seemed to be quite convalescent, when she was suddenly attacked with sinking spells and great weakness. Her family administered whiskey and beef tea, without much result. Here was a case where, according to common-sense ideas, "supporting treatment" was needed, and should have been given. Vigorous tonic treatment would have been administered by physicians of the regular school, and if the patient had died it would have been considered that all had been done for her that was possible. Her physician, howewer, being the editor of this journal, placed no confidence in the "supporting" treatment, but perversely sought the similar remedy. This proved to be Sulphur. It was given, with the most gratifying success, and the patient, after a long siege, was carried safely through the dangerous crisis.

Here, then, is an object lesson on the supreme importance of finding the true simillimum to any case.

Seek it, and find it, and the patient is rescued, to the gratification of the friends and the quiet self-satisfaction of the physician. Fail to find it, or neglect to make any serious effort to find it, and the patient slips through the doctor's fingers, to the grief of the family and the chagrin of the physician, as well as to the impairment of his reputation.

The responsibility resting upon the shoulders of the homœopathic physician is, therefore, very great. Only a conscientious man is fit to practice under this system, and he must have the approval of his own conscience if he would succeed, for he must have such stimulus to induce him to persevere in the onerous task of hunting for the simillimum in the vast labyrinth of our materia medica.

The ideal homœopathic prescription must cover every symptom which can be found in the present condition of the patient. If a drug be selected that does have this totality, there will be a response so prompt upon the part of the system when it is administered that it is absolutely bewildering to the homœopathist and hopelessly incredible to the skeptic. Rarely can we attain this ideal. Generally the proving of the drug will contain a part only of the symptoms, and sometimes these are not the most characteristic ones. Or, it may have one or two characteristic symptoms, but not those of lesser importance. Then the patient's recovery is more tardy. It is marked with more variations. It is less satisfactory to the physician.

Though it may not be possible to select the absolute simillimum in the majority of cases, yet the attainment of the totality should be the ideal which every physician should set before him and strive for with the earnestness that comes of the conviction that success will surely be achieved.

The artist who paints a great picture secures his success only because he has ever before him, in his mind's eye, a vivid ideal of perfection for which he strives. Without it his work would become commonplace, or even a failure. With it he wins fame and fortune.

In the same way must the homœopathic practitioner create in his own mind a great ideal of the simillimum. Emancipating himself from those theories of therapeutics which are founded upon the rational conception of pathological processes—conceptions which are ever changing—he must fix his mind upon, and consume his time in the study of the materia medica, that he may be ready promptly to apply it when the emergency arises. His reward—the amelioration of pain, the rescue from approaching death, the lustre to his name, the helping along the cause of a standard materia medica by verifying some symptoms and discovering the unreliability of others—ought to be a certain incentive to hard study and the rearing in his own mind of the ideal simillimum.

